# Characterization of Interleukin-15-Transpresenting Dendritic Cells for Clinical Use

**DOI:** 10.1155/2017/1975902

**Published:** 2017-07-13

**Authors:** J. M. J. Van den Bergh, E. L. J. M. Smits, M. Versteven, H. De Reu, Z. N. Berneman, V. F. I. Van Tendeloo, E. Lion

**Affiliations:** ^1^Laboratory of Experimental Hematology, Vaccine and Infectious Disease Institute (VAXINFECTIO), Faculty of Medicine & Health Sciences, University of Antwerp, Antwerp, Belgium; ^2^Center for Oncological Research Antwerp, Faculty of Medicine & Health Sciences, University of Antwerp, Antwerp, Belgium

## Abstract

Personalized dendritic cell- (DC-) based vaccination has proven to be safe and effective as second-line therapy against various cancer types. In terms of overall survival, there is still room for improvement of DC-based therapies, including the development of more immunostimulatory DC vaccines. In this context, we redesigned our currently clinically used DC vaccine generation protocol to enable transpresentation of interleukin- (IL-) 15 to IL-15R*βγ*-expressing cells aiming at boosting the antitumor immune response. In this study, we demonstrate that upon electroporation with both *IL-15* and *IL-15Rα*-encoding messenger RNA, mature DC become highly positive for surface IL-15, without influencing the expression of prototypic mature DC markers and with preservation of their cytokine-producing capacity and their migratory profile. Functionally, we show that IL-15-transpresenting DC are equal if not better inducers of T-cell proliferation and are superior in tumor antigen-specific T-cell activation compared with DC without IL-15 conditioning. In view of the clinical use of DC vaccines, we evidence with a time- and cost-effective manner that clinical grade DC can be safely engineered to transpresent IL-15, hereby gaining the ability to transfer the immune-stimulating IL-15 signal towards antitumor immune effector cells.

## 1. Introduction

Dendritic cells (DC) are the most professional antigen-presenting cells and the main orchestrators of our immune system [1]. Therefore, researchers have been implementing these cells as an immunotherapy in clinical trials to treat cancer patients for over 20 years now [2]. While DC-based vaccination has shown to be safe and effective in the battle against cancer, durable clinical responses remain scarce. For this reason, optimization of currently applied DC vaccines improving their immune-stimulating properties to generate superior antitumor immune responses is subject of intensive investigations [2–5].

Interleukin- (IL-) 15 was exclaimed as one of the most interesting immunotherapeutic agents for broad usage in cancer therapy [6, 7]. This nomination stems from the potent stimulatory effects of IL-15 on both the innate and the adaptive components of the immune system [8–11]. The superior immunostimulatory effects of IL-15 can be dedicated to the unique transpresentation mechanism it uses to transfer its signal to the effector cells of the immune system. Hereby, IL-15 binds to the *α*-moiety of its receptor, resulting in transpresentation of IL-15 to neighboring cells expressing the *βγ*-moiety of the IL-15 receptor on their membrane [12–14]. Since both natural killer (NK) cells and cytotoxic T-cells as main killer cells of the innate and adaptive immune system, respectively, display *βγ*-molecules on their membrane, it is postulated that IL-15 transpresentation can target these immune cells to increase the antitumor immune response [12, 13, 15]. In previous studies, we could indeed corroborate that the incorporation of the IL-15 transpresentation mechanism into currently used DC vaccines by means of mRNA electroporation increases their immunostimulatory properties towards both NK cells [16] and CD8^+^ T-cells [17]. More specifically, we demonstrated a DC-mediated enhancement of phenotypic NK cell activation and NK cell-mediated killing of tumor cells [16] and superior expansion of tumor-specific CD8^+^ T-cells [17].

Complementary to the exploitation of the immunostimulatory properties of DC vaccines, it is important for their clinical use that the incorporation of the IL-15 transpresentation mechanism (1) preserves the hallmark characteristics of the DC, (2) without dramatically increasing the cost and time to prepare the vaccine, and (3) guaranteeing product and patient safety. In this context, mRNA electroporation has already proven to be a feasible method to efficiently introduce molecules into DC, without introducing possibly noxious substances as with viral transfections [18–21]. Moreover, both mRNA encoding for immune-stimulating molecules, such as *IL-15*/*IL-15Rα*, and mRNA encoding for a specific antigen can be electroporated simultaneously into cells, circumventing the need of extra manipulating steps [22]. Transfection with mRNA has the additional safety advantage compared with DNA transfection that it cannot result in genomic integration and, therefore, will not permanently interfere with the normal function of human cells [21].

In this study, we describe how *IL-15* and/or *IL-15Rα* mRNA is implemented in a human clinical grade monocyte-derived DC vaccine protocol that is currently under investigation in three clinical trials (NCT01686334, NCT02649829, and NCT02649582) at our clinical trial facility at the Antwerp University Hospital, Belgium. We examined the effect of this manipulation on hallmark DC characteristics, that is, DC maturation phenotype, cytokine-producing profile, and lymph node-mediated migratory capacity. Acknowledging their superior antitumor function, we investigated their ability to induce T-cell proliferation and tumor antigen-specific T-cell activation.

## 2. Material and Methods

### 2.1. Ethics Statement and Cell Material

This study was approved by the Ethics Committee of the University of Antwerp (Antwerp, Belgium) under the Reference number 16/10/123. Experiments were performed using blood samples from anonymous donors provided by the Antwerp branch of the Red Cross Blood Transfusion Center (Mechelen, Belgium).

### 2.2. Messenger RNA (mRNA)

The human *OSP-IL-15* gene [23], which contains an optimized signal peptide (OSP) sequence before the IL-15-coding sequence, was generated into a pST1 vector by gene-ART (Life Technologies), putting it under the control of a T7 promoter and providing it with a poly(A) tail [24]. The human *IL-15Rα* gene was a kind gift of Dr. B. Weiner (University of Pennsylvania, Philadelphia, USA) and was subcloned into a pST1 vector. mRNA transcripts were generated using an mMessage mMachine T7 in vitro transcription kit (Life Technologies) according to the manufacturer's protocol.

### 2.3. Generation of IL-15 Designer DC

DC were generated as described previously [25, 26] with minor adaptations specific for the IL-15 designer DC. Briefly, positively selected CD14^+^ monocytes were differentiated into immature DC in the presence of IL-4 (20 ng/mL; Life Technologies) and granulocyte-macrophage colony-stimulating factor (800 U/mL; Gentaur) in Roswell Park Memorial Institute 1640 (RPMI; Invitrogen) supplemented with 2.5% human AB serum (SanBio). After 5 days, 20 ng/mL tumor necrosis factor-*α* (Gentaur) and 2.5 *μ*g/mL prostaglandin E2 (Pfizer, Puurs, Belgium) were added to induce maturation. Monocyte-derived DC (moDC) were harvested 40–44 hours later and electroporated by a time-constant (7 ms) pulse of 300 V using the Gene Pulser Xcell device (Bio-Rad) either without mRNA (mock EP DC), with 5 *μ*g *OSP-IL-15* mRNA (IL-15 EP DC), or with a combination of 5 *μ*g *OSP-IL-15* mRNA and 5 *μ*g *IL-15Rα* mRNA (IL-15/IL-15R*α* EP DC) in 200 *μ*L Opti-MEM reduced serum medium without phenol red (Life Technologies). Immediately after electroporation, DC were resuspended in prewarmed Iscove's Modified Dulbecco's Medium (IMDM; Invitrogen) + 10% fetal bovine serum (FBS) for further use.

### 2.4. Flow Cytometric Immunophenotyping

Phenotype of IL-15 designer DC was examined 4 h, 8 h, and 24 h after electroporation using combinations of fluorescein isothiocyanate- (FITC-) and phycoerythrin PE-conjugated monoclonal antibodies against CD14, CD40, CD70, CD80, CD86, CD209, HLA-DR, OX-40L (all BD), CD83 (Life Technologies), IL-15, and CCR7 (both R&D). Corresponding isotype staining was performed as negative control. 7-aminoactinomycin D (7-AAD; BD) was used to distinguish between viable and dead cells. All samples were measured on a FACScan flow cytometer (BD). Expression levels (delta mean fluorescence intensity (ΔMFI)) are expressed as relative levels compared to those of the corresponding mock EP DC, with ΔMFI representing subtraction of the MFI of the isotype control from the marker-specific MFI.

### 2.5. Cytokine Secretion Assays

Supernatant of DC cultures was examined 24 h after the electroporation for the presence of IL-4, IL-6, IL-10, IL-17A, IL-18, interferon- (IFN-) *α*2a, IFN-*γ*, and tumor necrosis factor- (TNF-) *α* using a custom-made U-plex kit for electrochemiluminescent detection (Meso Scale Discovery (MSD), Rockville, MD, USA) and performed according to the manufacturer's protocol. Data were analyzed on a SECTOR instrument (MSD) using MSD's Discovery Workbench software. Single IFN-*γ* analysis was quantified with a human IFN-*γ* ELISA kit (PeproTech) according to the manufacturer's protocol. Standards and samples were measured in duplicate and triplicate, respectively, in a 96-well flat bottom microplate (Nunc) on a Victor^3^ multilabel counter (PerkinElmer).

### 2.6. Migration Assay

The migratory potential of mock EP DC, IL-15 EP DC, and IL-15/IL-15R*α* EP DC was determined 4 h after electroporation by a chemotaxis assay using 24-well culture plates carrying polycarbonate membrane-coated Transwell™ permeable inserts (5 *μ*m pore size; Costar). The lower plate chambers were filled with 600 *μ*L IMDM + 10% FBS per well supplemented with the chemotactic CCR7 ligands CCL19 and CCL21 (R&D Systems) at an optimal concentration of 100 ng/mL for each agent. DC (1 × 10^5^ cells) were seeded on top of each transwell insert in a total volume of 100 *μ*L culture medium and allowed to migrate to the lower compartments for 180 min in a humidified 37°C/5% CO_2_ incubator (chemokine-driven migration). Parallel control experiments were conducted in the absence of CCL19 and CCL21 to assess the spontaneous cell migration (negative control) or by transferring all cells (1 × 10^5^) to the lower well in order to determine the maximum possible DC yield (positive control). Thirty minutes prior to harvest, 5 mM EDTA (Merck; Darmstadt, Germany) was added to the lower compartments to detach the transmigrated adherent cells. Finally, the cells from each lower well were collected, centrifuged, and concentrated to a final sample volume of 200 *μ*L. Cells were counted by flow cytometric analysis at a fixed flow rate during a defined time period of 60 sec (counts per minute (cpm)). DC migration was expressed using the following equation: %migrated cells = [(cpm_chemokine‐driven migration_ − cpm_negative control_)/cpm_positive control_] × 100.

### 2.7. Allogeneic Mixed Lymphocyte Reaction (Allo-MLR)

Thawed CD14-depleted peripheral blood lymphocytes (PBL) were labeled with 5,6-carboxyfluorescein diacetate succinimyl ester (CFSE; 5 *μ*M; Life Technologies) according to the manufacturer's instructions and used as responder cells in an allogeneic mixed lymphocyte reaction (allo-MLR) at a DC : responder cell ratio of 1 : 10. Specifically, 2 × 10^5^ allogeneic responder cells were cultured with 2 × 10^4^ mock EP DC, IL-15 EP DC, or IL-15/IL-15R*α* EP DC in 200 *μ*L IMDM supplemented with 10% FBS. Unstimulated PBL and a combination of phytohemagglutinin (PHA; 1 mg/mL; Sigma-Aldrich, Bornem, Belgium) and IL-2 (20 IU/mL; Immunotools) served as negative and positive controls, respectively. After 5 days, samples were stained with LIVE/DEAD® Fixable Aqua Stain (Life Technologies), CD3-PerCP-Cy5.5 (BD), CD4-APC-H7 (BD), and CD8PB (Life Technologies) and measured on a FACSAria II flow cytometer. CD4^+^ and CD8^+^ T-cell proliferation was assessed by quantifying the percentage of divided (CFSE-diluted) cells within the viable (LIVE/DEAD) CD3^+^CD4^+^ and CD3^+^CD8^+^ lymphocyte population, respectively.

### 2.8. Antigen Presentation Assay

The human cytotoxic T-cell clone (TCC) specific for the HLA-A∗0201-restricted epitope 126–134 of the Wilms' tumor 1 protein (WT1) [27] (kindly provided by Dr. C. Bonini, San Raffaele Scientific Institute, Milan, Italy) was maintained in IMDM/10% FBS with 60 IU/mL IL-2 (Immunotools, Friesoythe, Germany) and frozen in aliquots for immediate use upon thawing in functional assays. To evaluate their antigen-specific T-cell-activating capacity, IL-15 designer DC of HLA-A∗0201^−^ or HLA-A∗0201^+^ donors were loaded with 10 *μ*g/mL WT1_126_ peptide (RMFPNAPYL; JPT Peptide Technologies) and cocultured with the TCC at DC:TCC ratios of 1 : 10, 1 : 20, and 1 : 40 in IMDM supplemented with 2% human AB serum in triplicate in 96-well round bottom microplates. Cocultures of the TCC with WT1_126_ peptide-pulsed T2 cells (HLA-A∗0201^+^, WT1^−^ cell line; kindly provided by Dr. Pierre Van der Bruggen, Ludwig Institute for Cancer Research, Brussels, Belgium) served as positive controls. To determine the levels of non-antigen-specific IFN-*γ* production, the TCC was cultured alone and cultured with non-peptide-pulsed stimulator cells. After overnight coculture, supernatants were collected and cryopreserved at −20°C for IFN-*γ* quantification.

### 2.9. Statistical Analysis

Flow cytometry data were analyzed using FlowJo version 10.0.6 (Tree Star, Ashland, OR, USA). GraphPad Prism 5 software (GraphPad, San Diego, CA, USA) was used for graphing and statistical calculations. Statistical analysis was performed using the repeated measures one-way or two-way analysis of variance with Bonferroni post hoc test, where appropriate. The results were considered statistically significant when *p* < 0.05.

## 3. Results

### 3.1. The Mature DC Phenotype Is Unaffected upon *IL-15* and *IL-15Rα* mRNA Electroporation

**Figure 1:**
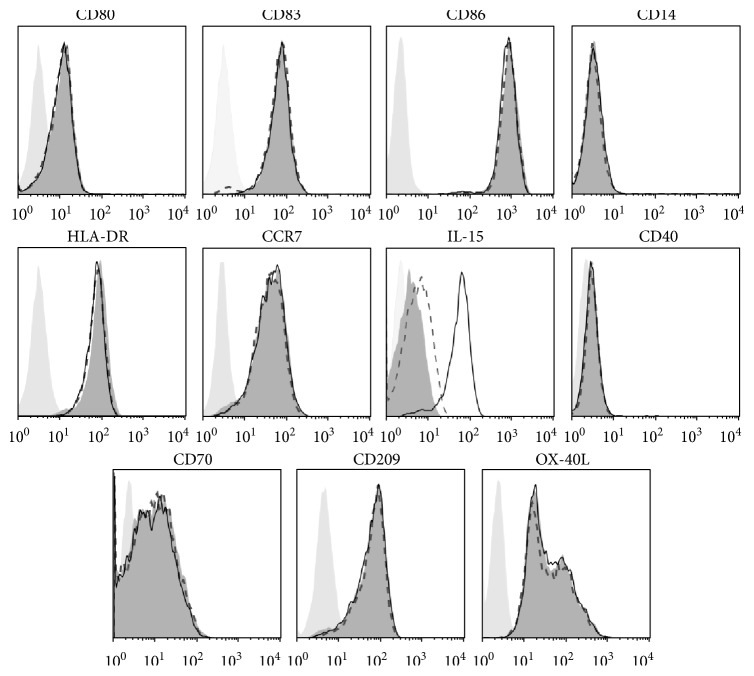
Matured phenotype of IL-15 designer DC. Surface expressions of CD80, CD83, CD86, CD14, HLA-DR, CCR7, IL-15, CD40, CD70, CD209, and OX-40L on IL-15 EP DC (dashed-lined histogram) or IL-15/IL-15R*α* EP DC (thin-lined black histogram) were compared 4 h after electroporation with mock EP DC (dark-grey-filled histogram) and isotype controls (light-grey-filled histogram). Histogram overlays are shown for one representative donor out of six independent donors. CCR7, C-C chemokine receptor type 7; HLA, human leukocyte antigen; IL, interleukin.

The manipulation of clinical grade mature DC with *IL-15* and *IL-15Rα* mRNA electroporation resulted in high IL-15 surface expression (Figure 1; [16]) but had no effect on other phenotypic DC markers. More detailed, 4 h after electroporation, the monocyte marker CD14 was absent on all DC types, while the prototypic DC maturation markers CD80, CD83, and CD86 were equally high expressed on the membrane of mock EP DC (dark-grey-filled histogram), IL-15 EP DC (dashed-lined histogram), and IL-15/IL-15R*α* EP DC (thin-lined black histogram) as compared to the corresponding isotype (light-grey-filled histogram) (Figure 1). Also, no differences could be detected on all other DC markers tested, human leukocyte antigen- (HLA-) DR, CD40, CD209, CCR7, CD70, and OX-40L (Figure 1). Throughout time (4 h, 8 h, and 24 h postelectroporation), the *IL-15/IL-15Rα* mRNA electroporation of the DC vaccine had no effect on the mature DC phenotype (Supplemental Figure 1 available online at https://doi.org/10.1155/2017/1975902).

### 3.2. Cytokine Secretion Profile of IL-15 Designer DC

Depicted in Table 1, levels of typical T helper- (Th-) 2 cytokines IL-4 and IL-10 and the typical Th-17 cytokine IL-17 remained below the detection limits, irrespective of the applied electroporation. Secretion of the proinflammatory cytokines IFN-*α*2a, TNF-*α*, IL-6, and IL-18 was not affected by the introduction of IL-15 transpresentation. Low levels of IFN-*γ* (<55 pg/mL) were detected in supernatants of both IL-15 EP DC (*p* < 0.01) and IL-15/IL-15R*α* EP DC (*p* < 0.01) as compared to mock EP DC in which IFN-*γ* concentrations remained below the detection limit (DL = 24 pg/mL; Table 1).

### 3.3. IL-15 Designer DC Exert Potent Migratory Capacity

**Figure 2:**
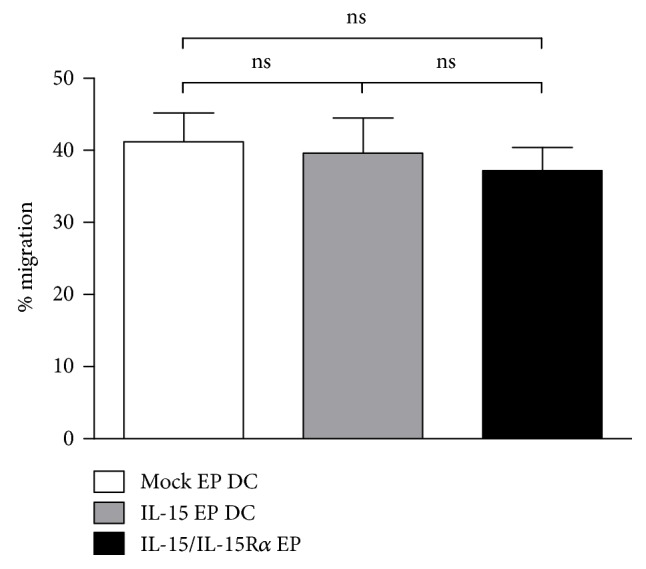
Migratory capacity of IL-15 designer DC. Bars depict the mean percentage (±SEM; *n* = 6) CCL19/CCL21-mediated migration of mock EP DC (white bar), IL-15 EP DC (grey bar), and IL-15/IL-15R*α* EP DC (black bar) 4 h after electroporation in a 3 h transwell chemotaxis assay. DC migration was calculated according to the equation specified in the Material and Methods. DC, dendritic cells; EP, electroporation; cpm, counts per minute; IL, interleukin; IL-15R*α*, interleukin-15 receptor alpha; SEM, standard error of mean.

The hallmark IL-4 moDC C-C chemokine receptor type 7 (CCR7) is equally high expressed on IL-15 EP DC and IL-15/IL-15R*α* EP DC (Figure 1). To confirm functionality, we evaluated the migratory potential of the different DC types towards the lymph node-recruiting CCR7 ligands CCL19 and CCL21. Migration of IL-15 EP DC (39.6 ± 1.8%; mean ± SEM) and IL-15/IL15R*α* EP DC (37.2 ± 1.2%) did not differ significantly with mock EP DC migration (41.2 ± 1.5%; Figure 2).

### 3.4. IL-15 Designer DC Can Have Higher T-Cell-Proliferating Capacity

**Figure 3:**
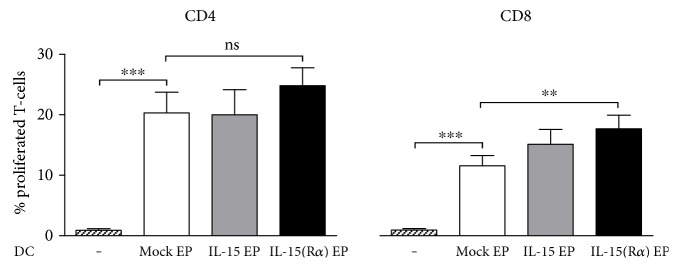
T-cell stimulatory capacity of IL-15 designer DC in an allo-MLR. Bar graphs depict proliferation of PBL upon 5-day coculture with mock EP DC (white bar), IL-15 EP DC (grey bar), or IL-15/IL-15R*α* EP DC (black bar) at a 10 : 1 T-cell/DC ratio. Cocultures were analyzed for CD4^+^ T-cell and CD8^+^ T-cell proliferation within the viable CD3^+^ T-cell population by flow cytometry. Data are shown as mean (±SEM) for 4 independent donors. ^∗∗^*p* < 0.01; ^∗∗∗^*p* < 0.001, repeated measures one-way ANOVA with Bonferroni post hoc test. ADC, dendritic cells; IL, interleukin; IL-15R*α*, interleukin-15 receptor alpha; ns, not significant; PBL, peripheral blood lymphocytes; SEM, standard error of the mean.

As key characteristic of DC, we assessed the IL-15 designer DC-mediated proliferation of both CD4^+^ and CD8^+^ T-cells in an allogeneic mixed lymphocyte reaction. Nonstimulated lymphocytes served as negative control (PBL; Figure 3). Five-day coculture of IL-4 moDC (mock EP DC) with allogeneic lymphocytes resulted in significant proliferation of both CD4^+^ (*p* < 0.001) and CD8^+^ T-cells (*p* < 0.001). DC transfected with *IL-15* mRNA only (IL-15 EP DC) did not induce improved T-cell proliferation, while DC electroporated with the combination of *IL-15* and *IL-15Rα* mRNA (IL-15/IL-15R*α* EP DC) exerted significant higher CD8^+^ T-cell proliferation (*p* < 0.01) and higher CD4^+^ T-cell proliferation (*p* = 0.0781) relative to mock EP DC.

### 3.5. IL-15 Designer DC Provide Superior WT1-Specific T-Cell Activation

**Figure 4:**
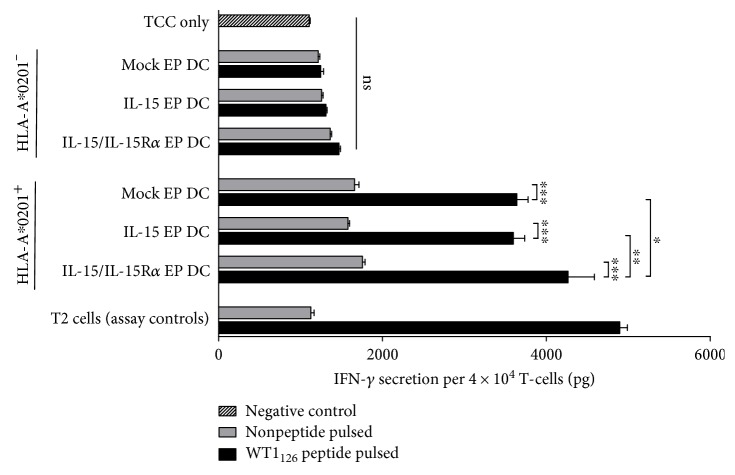
Stimulation of WT1-specific CD8^+^ T-cells by IL-15 designer DC. A WT1_126–134_-specific HLA-A∗0201-restricted CD8^+^ T-cell clone was stimulated with mock EP DC, IL-15 EP DC, or IL-15/IL-15R*α* EP DC from HLA-A∗0201^+^ (*n* = 3) or HLA-A∗0201^−^ donors (*n* = 3) or with control T2 cells at a 10 : 1 TCC/stimulator cell ratio or left unstimulated (TCC only). To assess antigen specificity, stimulator cells were either peptide pulsed (black bars) or left unloaded (grey bars). After overnight coculture, cell-free supernatants were harvested and analyzed for IFN-*γ* using ELISA (mean ± SEM). ^∗^*p* < 0.05; ^∗∗^*p* < 0.01; ^∗∗∗^*p* < 0.001, repeated measures one-way ANOVA with Bonferroni post hoc test. DC, dendritic cells; HLA, human leukocyte antigen; IFN, interferon; IL, interleukin; IL-15R*α*, interleukin-15 receptor alpha; ns, not significant; SEM, standard error of the mean; TCC, T-cell clone.

The capacity of IL-15 designer DC to present tumor-specific antigen was assessed in an HLA-restricted WT1-specific T-cell model (Figure 4). WT1_126_ peptide presented by DC from HLA-A∗0201^+^ donors triggered high amounts of IFN-*γ* by a WT1_126–134_-specific CD8^+^ T-cell clone after overnight coculture at different DC:TCC ratios (1 : 10, 1 : 20, 1 : 40). Stimulation with IL-15/IL-15R*α* EP DC (4266 ± 224 pg/4 × 10^4^ TCC; mean ± SEM, *n* = 3), but not with IL-15 EP DC (3598 ± 134 pg/4 × 10^4^ TCC) induced significant higher IFN-*γ* secretion compared to coculture with their mock-transfected counterparts (mock EP DC, 3639 ± 122 pg/4 × 10^4^ TCC) at all ratios tested (1 : 10, Figure 4; 1 : 20 and 1 : 40, data not shown). As a control, DC from HLA-A∗0201^−^ donors, either unloaded or peptide pulsed, did not induce TCC IFN-*γ* above background (TCC only; Figure 4). Likewise, TCC stimulated with non-peptide-pulsed DC from HLA-A∗0201^+^ donors showed no non-specific-elevated IFN-*γ* secretion levels.

## 4. Discussion

Due to the pleiotropic attribute of IL-15 to stimulate both the innate and the adaptive arm of the immune system and growing preclinical data on IL-15-mediated antitumor immunity, IL-15 was categorized as one of the immunotherapeutic agents with high potential for broad usage in cancer therapy [6, 7]. In line with these observations, the antitumor potency of systemic IL-15 administration was further investigated in both animal models [28, 29] and in the first-in-human clinical trial [8]. Although systemic delivery of IL-15 resulted in efficient activation of antitumor responses, this was accompanied with substantial systemic cytotoxicity, particularly when administered on a daily basis [8, 28, 29]. Together with some early clinical disappointments with systemic cytokine-based immunotherapy, including IL-15, pharmaceutical companies are not inclined to produce clinical grade therapeutic cytokines anymore [30, 31]. Additionally, the half-life of IL-15 is less than one hour, limiting its bioactivity in vivo after systemic delivery. By binding to IL-15R*α*, which occurs in the so-called IL-15 transpresentation process, the half-life and stability of IL-15 can be prolonged [32, 33].

Benefiting from its immunostimulatory properties, while evading systemic delivery of clinical grade IL-15, a different approach of IL-15 transfection in immune-competent cells was assessed in this study, effectuating in situ production, secretion, and transpresentation of IL-15. The goal of this study was to evaluate a clinically feasible protocol generating IL-15-secreting and IL-15-transpresenting cells by simultaneously electroporating *IL-15* and *IL-15Rα*-encoding mRNA into DC. From a clinical perspective, it is more feasible to obtain clinical grade *IL-15* and *IL-15Rα* mRNA for mRNA-based transfection (e.g., through electroporation) [34] than the purified proteins, circumventing the hurdle of the only scarcely available clinical grade proteins IL-15 and IL15R*α*. With this innovative designer DC-based strategy, we aimed to develop highly potent immune-stimulatory DC for future use in DC vaccination trials.

In the perspective of optimization of existing DC vaccine preparations, manipulations are to be evaluated for their safety and immune-stimulatory characteristics. In our WT1-targeted DC vaccination trials for acute myeloid leukemia (NCT01686334), glioblastoma (NCT02649582), and mesothelioma patients (NCT02649829), viability, DC morphology, phenotype (CD86, HLA-DR, CCR7), and positive migration are the most important release criteria before the DC vaccine can be administered to patients. With this study, we can confirm that the incorporation of both *IL-15* and *IL-15Rα* mRNA via electroporation into the DC vaccine does not interfere with these criteria, while IL-15—responsible for superior in vitro T-cell stimulation and previously demonstrated NK cell activation [16], is highly presented on the membrane of the DC product. In addition, we show that this manipulation has also no effect on the more elaborate DC marker profile (CD14, CD40, CD70, CD80, CD83, CD209, and OX-40L). In contrast to our mRNA transfection technique, Tourkova and colleagues showed that adenoviral transduction of the IL-15 gene into human moDC resulted in elevated expression of costimulatory molecules on the DC membrane, enhanced IL-12 expression by the DC, and the ability to induce T-cell proliferation [35]. It is suggested that the observed effects were caused by signaling through the *βγ*-moiety of the IL-15 receptor, which can be presented on both T-cells and monocytes [36]. Using *IL-15*/*IL-15Rα* mRNA transfection, we demonstrate that there is no significant influence on the DC phenotype, migratory capacity, nor cytokine production as compared to mock-electroporated DC. This might imply that our IL-15-transpresenting DC do not, or only in low levels, express the *β*- or *γ*-moiety of the IL-15 receptor. These discrepancies could be explained by the differences in DC vaccine preparation, such as delivery method of IL-15 (mRNA electroporation versus transduction via adenoviral gene integration) and time point of IL-15 delivery (immature versus mature DC stage). Furthermore, as a hallmark of DC, our IL-15-conditioned DC preserve the capacity to induce allogeneic T-cell proliferation, with a slight increase in CD8^+^ T-cell proliferation when both *IL-15* and *IL-15Rα* mRNA are introduced in DC. This indicates that IL-15 transpresentation can have immune-stimulating effects towards CD8^+^ T-cells [37, 38]. This is further evidenced by superior activation of both the WT1-specific CD8^+^ T-cell clone used in this paper and antigen-specific CD8^+^ T-cells from hematological cancer patients [17] after stimulation with IL-15-transpresenting DC.

Altogether, we report on the development of clinically applicable designer DC implementing the IL-15 transpresentation mechanism into IL-4 moDC while maintaining hallmark properties of the DC. Since mRNA electroporation is broadly accepted to introduce tumor antigens into DC, in situ cotransfection with immune-stimulatory molecules like *IL-15* and *IL-15Rα* mRNA can be easily performed in one electroporation step, avoiding the need of time- and cost-consuming manipulations [22, 39]. With only minor modifications to the DC generation protocol, designer DC gain the ability to transfer the immune-stimulatory signal of IL-15 in a safe nonsystemic way to IL-15R*βγ*-expressing cells (e.g., NK cells and T-cells) in favor of strong (antigen-specific) antitumor immune responses.

## Supplementary Material

Supplemental Figure 1. Matured phenotype of IL-15 designer DC at multiple time points after electroporation. IL-15 EP DC (grey line) and IL-15/IL-15Rα EP DC (black line) were evaluated flow cytometrically for surface expression of CD80, CD83, CD86, HLA-DR, CCR7, OX-40L, CD70 and CD209 at 4h, 8h and 24h after electroporation. Results are depicted as mean percentage (± SEM; n⁼6) membrane marker expression relative to mock EP DC as follows: (dMFI IL-15 EP DC or IL-15/IL-15Rα EP DC/dMFI mock EP DC) × 100, with dMFI representing subtraction of the MFI of the isotype control from the membrane marker-specific MFI. Abbreviations: CCR7; C-C chemokine receptor type 7, dMFI; delta mean fluorescence intensity, HLA; human leukocyte antigen, IL; interleukin, IL-15Rα; interleukin-15 receptor alpha, SEM; standard error of the mean.

## Figures and Tables

**Table 1:** Cytokine secretion (±SEM) in 24 h washed-out supernatant by IL-15 designer DC. DC: dendritic cell; DL: detection limit; EP: electroporated; IFN: interferon; IL: interleukin; SEM: standard error of mean; TNF: tumor necrosis factor. ^∗∗^*p* < 0.01 (compared to mock EP DC) (*n* = 6).

	Mock EP DC	IL-15 EP DC	IL-15/IL-15R*α* EP DC
	(pg/mL)	(pg/mL)	(pg/mL)
IFN-*α*2a	0 ± 0	0 ± 0	0 ± 0
IFN-*γ*	19 ± 2 < DL	54 ± 3^∗∗^	52 ± 3^∗∗^
TNF-*α*	62 ± 3	75 ± 3	74 ± 5
IL-4	0 ± 0	0 ± 0	0 ± 0
IL-6	176 ± 21	167 ± 18	167 ± 19
IL-10	0 ± 0	0 ± 0	0 ± 0
IL-17	0 ± 0	0 ± 0	0 ± 0
IL-18	113 ± 80	103 ± 73	104 ± 74
