# Supplementary figures and images for: Characterization of Interleukin-15-Transpresenting Dendritic Cells for Clinical Use
Source: J Immunol Res. 2017 Jul 13;2017:1975902. doi: 10.1155/2017/1975902 (PMC5530419; doi:10.1155/2017/1975902)

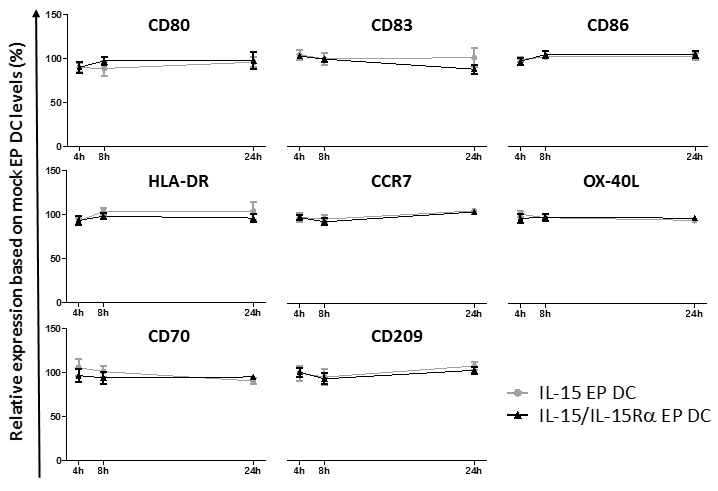

Supplement: Supplementary file 1 — Supplemental Figure 1. Matured phenotype of IL-15 designer DC at multiple time points after electroporation. IL-15 EP DC (grey line) and IL-15/IL-15Rα EP DC (black line) were evaluated flow cytometrically for surface expression of CD80, CD83, CD86, HLA-DR, CCR7, OX-40L, CD70 and CD209 at 4h, 8h and 24h after electroporation. Results are depicted as mean percentage (± SEM; n⁼6) membrane marker expression relative to mock EP DC as follows: (dMFI IL-15 EP DC or IL-15/IL-15Rα EP DC/dMFI mock EP DC) × 100, with dMFI representing subtraction of the MFI of the isotype control from the membrane marker-specific MFI. Abbreviations: CCR7; C-C chemokine receptor type 7, dMFI; delta mean fluorescence intensity, HLA; human leukocyte antigen, IL; interleukin, IL-15Rα; interleukin-15 receptor alpha, SEM; standard error of the mean. [file 1975902.f1.tif]
